# Enhanced Osseointegration by the Hierarchical Micro-Nano Topography on Selective Laser Melting Ti-6Al-4V Dental Implants

**DOI:** 10.3389/fbioe.2020.621601

**Published:** 2021-01-07

**Authors:** Tianyu Shu, Yuchen Zhang, Guo Sun, Yang Pan, Gang He, Yilong Cheng, Ang Li, Dandan Pei

**Affiliations:** ^1^Key Laboratory of Shaanxi Province for Craniofacial Precision Medicine Research, College of Stomatology, Xi’an Jiaotong University, Xi’an, China; ^2^State Key Laboratory of Military Stomatology, School of Stomatology, The Fourth Military Medical University, Xi’an, China; ^3^Frontier Institute of Science and Technology, Xi’an Jiaotong University, Xi’an, China; ^4^School of Chemistry, Xi’an Jiaotong University, Xi’an, China; ^5^Department of Periodontology, College of Stomatology, Xi’an Jiaotong University, Xi’an, China; ^6^Department of Prosthodontics, College of Stomatology, Xi’an Jiaotong University, Xi’an, China

**Keywords:** selective laser melting, dental implant, surface, hierarchical micro-nano topography, osseointegration

## Abstract

Currently, selective laser melting (SLM) has been thriving in implant dentistry for on-demand fabricating dental implants. Based on the coarse microtopography of SLM titanium surfaces, constructing nanostructure to form the hierarchical micro-nano topography is effective in enhancing osseointegration. Given that current nanomodification techniques of SLM implants, such as anodization and hydrothermal treatment, are facing the inadequacy in costly specific apparatus and reagents, there has been no recognized nanomodified SLM dental implants. The present study aimed to construct hierarchical micro-nano topography on self-made SLM dental implants by a simple and safe inorganic chemical oxidation, and to evaluate its contribution on osteoblastic cells bioactivity and osseointegration. The surface chemical and physical parameters were characterized by FE-SEM, EDS, profilometer, AFM, and contact angle meter. The alteration on bioactivity of MG-63 human osteoblastic cells were detected by qRT-PCR. Then the osseointegration was assessed by implanting implants on the femur condyle of New Zealand Rabbits. The hierarchical micro-nano topography was constituted by the microrough surface of SLM implants and nanoneedles (diameter: 20∼50 nm, height: 150∼250 nm), after nanomodifying SLM implants in 30% hydrogen peroxide and 30% hydrochloride acid (volume ratio 1:2.5) at room temperature for 36 h. Low chemical impurities content and high hydrophilicity were observed in the nanomodified group. Cell experiments on the nanomodified group showed higher expression of mitophagy related gene (PINK1, PARKIN, LC3B, and LAMP1) at 5 days and higher expression of osteogenesis related gene (Runx2 and OCN) at 14 days. In the early stage of bone formation, the nanomodified SLM implants demonstrated higher bone-to-implant contact. Intriguingly, the initial bone-to-implant contact of nanomodified SLM implants consisted of more mineralized bone with less immature osteoid. After the cessation of bone formation, the bone-to-implant contact of nanomodified SLM implants was equal to untreated SLM implants and marketable TixOs implants. The overall findings indicated that the inorganic chemical oxidized hierarchical micro-nano topography could enhance the bioactivity of osteoblastic cells, and consequently promote the peri-implant bone formation and mineralization of SLM dental implants. This study sheds some light on improvements in additive manufactured dental implants.

## Introduction

Globally, over 275 million people were suffering from edentulous malady (Vos et al., 2016). As a routine procedure for replacing missing teeth, implant denture had demonstrated significant oral functional rehabilitation and excellent long-term prognosis (Bosshardt et al., 2017; Buser et al., 2017). Current dental implants were manufactured by traditional metal fabrication processes like forging, casting, hot rolling, and machining (Andani et al., 2014). Such implants were limited by fixed specification on macro design and dimension, that sometimes necessitated costly and invasive bone augmentation surgery (Oliveira and Reis, 2019). For on-demand dental implants fabrication, the selective laser melting (SLM), as one of additive manufacturing techniques, had shown great facilitations on product customization and cost control (Trevisan et al., 2018). Besides, due to the higher microroughness of SLM titanium surfaces could accommodate cell attachment and bone-to-implant mechanical anchorage (Dos Santos et al., 2019; Liu et al., 2019), SLM dental implants had demonstrated similar even better osseointegration than traditional dental implants (Oliveira and Reis, 2019). However, titanium and its alloys were bioinert (Bhargav et al., 2018). Modifying SLM dental implants to accelerate osseointegration was still an important issue.

The hierarchical micro-nano surface topography, due to its similarity with the multi-ordered structure of human bone, had become a better choice for intrabony biomaterials (Cui et al., 2019; Zhang et al., 2019). It had been proven that the bioactivity of SLM dental implants, including *in vitro* mesenchymal stem cells adherence, proliferation, differentiation, and *in vivo* osseointegration could be improved by the hierarchical micro-nano topography (Ferraris et al., 2011, 2019; Gulati et al., 2017; H. Wang et al., 2018; Yu et al., 2018). An up to date research had verified that the osteogenesis promoting effect of nanomodified SLM implants was strongly related to the integrin α2-PI3K-AKT signaling axis (Zheng et al., 2020). Today, SLM titanium surfaces could be nanomodified *via* anodization (Gulati et al., 2017), hydrothermal (H. Wang et al., 2018; Yu et al., 2018), or inorganic chemical treatments (Ferraris et al., 2011, 2019). These surface modifications enabled additional nanotopography while preserving the nature microroughness of SLM surfaces, and consequently constituted the hierarchical micro-nano topography. However, both anodization and hydrothermal treatment were under the need of specific apparatus and reagents. The current inorganic chemical treatment was also facing the limitation of dangerous hydrofluoride acid. There was no certified nanotextured SLM dental implant product yet.

In this study, we introduced the osseointegration of nanotextured SLM implants modified by an improved inorganic chemical oxidation method. Such protocol could be used to construct the hierarchical micro-nano topography on the surface of self-made SLM Ti-6al-4V dental implants in a simple and safe manner. For certifying the validity of nanomodification, we characterized the topography, roughness, chemical components, and wettability of modified and untreated SLM titanium surfaces. *In vitro* osteogenesis, mitochondrial dynamics and *in vivo* osseointegration were examined by MG-63 human osteoblastic cell culturing and animal experiments. A commercially available SLM dental implant (TixOs, Leader Implants, Italy) was used as the control group due to its favorable clinical case reports (Mangano et al., 2012, 2017). As the result, for SLM implants with hierarchical micro-nano topography, promoted bioactivity of osteoblastic cells was found at the gene expression level, and improved peri-implant bone formation and mineralization were proven by bone histomorphometric analysis. In brief, this paper tried to facilitate the development of hierarchical micro-nano topography for future SLM dental implants.

## Materials and Methods

### SLM Implants Fabrication and Surface Modification

The raw material of SLM was Ti-6Al-4V powder in 25∼45 μm diameter. At first, the computer-aided design (CAD) model of an implant with 3.75 mm diameter and 6.0 mm height was designed in Solidworks 2015 and then exported to the printer (SLM-150, Hanbang, Guangzhou, China) in the STL format. Key SLM parameters include laser power (300 W), scanning rate (500 mm/s), line width (60 μm), and layer thickness (50 μm). Finally, SLM implants were ultrasonic cleaned in distilled water for 15 min, as the SLM-UT (untreated) group in this study. Half of SLM implants were selected randomly to be nanomodified. These implants were immersed in a mixture solution (volume ratio 1:2.5) of 30% hydrogen peroxide and 30% hydrochloride acid at room temperature. After 36 h, these nanomodified implants were rinsed with distilled water thoroughly, as the SLM-CO (chemical oxidized) group. For cell experiments, untreated and nanomodified SLM Ti-6Al-4V chips (10 mm × 10 mm × 1 mm) were also fabricated by the same working condition of SLM-UT and SLM-CO groups.

### Surface Characterization

The surface topography was characterized by a field emission scanning electronic microscope (FE-SEM; Hitachi S-4800, Japan). The surface arithmetical mean height (S_*a*_) at micro and nano scale were detected, respectively, by a 3D profilometer (PS50, NANOVEA, United States) in a 400 μm × 400 μm area and an atomic force microscope (AFM; 5500, Agilent, United States) in a 2.5 μm × 2.5 μm area. The surface chemical composition was analyzed by an energy-dispersive X-ray spectrometer (EDS; EDAX, United States). The surface wettability with distilled water in the air was detected by an optical contact angle meter (DSA100, Kruss, Germany).

### Cell Experiments

#### MG-63 Cells Seeding and Culturing

The SLM-UT and SLM-CO chips were sterilized by ultraviolet irradiation for 30 min and placed individually into the 24-well plate (Corning, United States). The human MG-63 osteoblastic cells (ATCC CRL1427, United States) were cultured in Dulbecco’s Modified Eagle Medium (Gibco, United States) supplemented with 10% Fetal bovine serum (Gibco, United States) and 0.5% Anti-anti (Gibco, United States) at a primary density of 2 × 10^4^ cells/well. Cells were incubated in a humidified atmosphere of 5% CO_2_ at 37°C. The culture medium was renewed every 2 days.

#### MTT Assay

The cell proliferation level was evaluated by the MTT [3-(4,5-Dimethylthiazol-2-yl)2,5-diphenyltetrazolium bromide] assay at 1, 3, and 5 days after cell seeding. At each time point, SLM-UT and SLM-CO chips were transferred to new wells and incubated at 37°C with a culture medium containing MTT (0.5 mg/ml) for 4 h in dark. Next, the intracellular purple formazan product of each sample was dissolved using 375 μL dimethyl sulfoxide (DMSO). After smoothly oscillated for 15 min, 150 μL supernate in each sample was transferred to a 96-well plate (Corning, United States). The absorbance at 570 nm of each well was quantified by a spectrophotometer (Multiskan FC, Thermo, United States).

#### Alizarin Red Staining

After cell seeding for 14 days, SLM-UT and SLM-CO chips were washed with distilled water twice and fixed in 3.7% formaldehyde at room temperature for 10 min. To make mineralized nodules visualized, the chips were immersed in a 2% Alizarin red S solution (pH 4.2) at 37 °C and washed with distilled water after 30 min.

#### Quantitative Real-Time PCR

The Quantitative Real-time PCR (qRT-PCR) assay was performed to study the effect of nanomodified SLM implants on the expression of genes related to osteogenesis and mitochondrial dynamics. After seeding MG-63 cells for 5 and 14 days, respectively, total RNA was extracted by Trizol reagent (Invitrogen, United States). The quantity and quality of the RNA were measured by a nano spectrophotometer (DS-11, DeNovix, United States). Then, cDNA was synthesized through a reverse transcription premix kit (AG11706, Accurate Biotechnology, China). The glyceraldehyde-3-phosphate dehydrogenase (GAPDH) was used as the housekeeping gene. For investigating the mitochondrial dynamics, the relative expressions of peroxisome proliferator-activated receptor-γ coactivator-1 α and β (PGC-1α and PGC-1β), DNA polymerase γ (POLγ), mitochondrial transcription factor A (TFAM), mitofusin-1 (MFN1), mitofusin-2 (MFN2), dynamin-related protein-1 (DRP1), mitochondrial fission protein-1 (FIS1), phosphatase-and-tensin homolog-induced putative kinase 1 (PINK1), PARKIN, microtubule-associated protein-1 light-chain 3-B (LC3B), and lysosomal-associated membrane protein 1 (LAMP1) were measured at 5 days. For comparing the osteogenesis effect, the relative expressions of Runt-related transcription factor 2 (Runx2) and osteocalcin (OCN) were measured at 14 days. Primer sequences were listed in Table 1 and synthesized by Sangon Biotech (Shanghai, China). The qRT-PCR procedure was performed by using SYBR green PCR reaction mix kit (AG11701, Accurate Biotechnology, China) on the real-time PCR system (AB 7500, Applied Biosystems, United States). Results were calculated using the 2^–ΔΔ*Ct*^ method and presented as fold regulations relative to the control.

**TABLE 1:** Primer sequences used in quantitative real-time PCR.

**Gene**	**Forward sequence**	**Reverse sequence**
GAPDH	GCACCGTCAAGGCTGAGAAC	TGGTGAAGACGCCAGTGGA
Runx2	AGGCAGTTCCCAAGCATTTCATCC	TGGCAGGTAGGTGTGGTAGTGAG
OCN	CGCTACCTGTATCAATGGCTGG	CTCCTGAAAGCCGATGTGGTCA
PGC-1α	CAGAGAGTATGAGAAGCGAGAG	AGCATCACAGGTATAACGGTAG
PGC-1β	TTCAGACAGAACGCCAAGCATCC	CAGCACCTCGCACTCCTCAATC
POLγ	GCACCGTCAAGGCTGAGAAC	CAAGTCATTCAGACCCAGCTTGTA
TFAM	GAGGTGGTTTTCATCTGTCTTGG	CAACGCTGGGCAATTCTTCT
MFN1	GTGGCAAACAAAGTTTCATGTG	CACTAAGGCGTTTACTTCATCG
MFN2	GTGCTTCTCCCTCAACTATGAC	ATCCGAGAGAGAAATGGAACTC
DRP1	GAGATGGTGTTCAAGAACCAAC	CAATAACCTCACAATCTCGCTG
FIS1	AGTACGCCTGGTGCCTGGTG	GCTGTTCCTCCTTGCTCCCTTTG
PINK1	GGACGCTGTTCCTCGTTA	ATCTGCGATCACCAGCCA
PARKIN	AACCGGTACCAGCAGTATGG	TTCGCAGGTGACTTTCCTCT
LC3B	AGCAGCATCCAACCAAAA	CTGTGTCCGTTCACCAACAG
LAMP1	GTGTCTGCTGGACGAGAACA	TAGCCTGCGTGACTCCTCTT

### Animal Experiment

#### Animal Surgical Operation

A rational animal model was vital for an animal experiment. The bone structure of rabbit femoral condyle was similar to that of human alveolar bone, which was spongy cancellous bone wrapped by thin cortical bone (Wu et al., 2019). Hence, the New Zealand Rabbit was widely used in evaluating dental implant functions (Wancket, 2015). The experiment protocol was approved by The Laboratory Animal Care and Welfare Committee, Fourth Military Medical University (No. 2018-K9-020). Male New Zealand Rabbits (weight 3.25 ± 0.25 kg) were obtained from the Junxing Biomedical (Xi’an, China), and were housed in a temperature-controlled room with 12 h alternating light-dark cycle at The Laboratory Animal Center, State Key Laboratory of Military Stomatology (Xi’an, China). Fodder and water were given ad labium. All rabbits were acclimatized for 7 days before the operation. For anesthesia, the xylazine hydrochloride injection (Huamu Veterinary Medicine, Jilin, China) at 0.1 mL/kg were intramuscularly injected, followed by a slow intravenous injection of 3% pentobarbital sodium (Sigma, United States) at 0.8 mL/kg. Benzylpenicillin sodium (Harbin Pharmaceutical Group Holding, Harbin, China) at 800,000 U was intramuscularly injected as a prophylactic antibiotic. Rabbits were then transferred to a thermostatic operating table to avoid hypothermia. We used a parapatellar incision to expose the outside surface of the femoral condyle in a minimal wound (Supplementary Figure 1). The planting hole (diameter: 3.75 mm, depth: 6.0 mm) was prepared by intermittent drilling at a speed of 800 rpm and rinsed with normal saline at 4°C. After douching implant holes with normal saline thoroughly, implants were rotated into holes slowly. Then the surgical field was irrigated by 80,000 U gentamicin and sutured in layers. During post-operation 5 days, all rabbits received wound disinfection with iodine and intramuscularly injecting benzylpenicillin sodium 800,000 U per 24 h.

#### Specimen Harvesting

Specimens were harvested at post-operation 2, 4, and 8 weeks. Each time point included 10 SLM-UT, 10 SLM-CO, and 5 TixOs implants. Rabbits were euthanized with a lethal dose of pentobarbital sodium. Femur condyles of all rabbits were cut off carefully and then trimmed as implant-containing bone blocks (1.5 cm × 1.5 cm × 0.6 cm) by a bone tissue cutting machine (312, EXAKT, Germany). Then, all implant-containing bone blocks were immediately fixed in 4% formaldehyde for at least 72 h.

#### Micro CT

A micro X-ray 3D imaging system (Y.Cheetah, YXLON, Germany) was used to scanning the peri-implant bone structure, in the regime 55.6 μA and 90 kV. The isotropic resolution of scanning is 9 μm. Raw data were imported into VG Studio MAX 3.0.2 (Volume Graphics, Germany) and reconstructed *via* beam hardening correction and ring artifact elimination. The region of interest (ROI) was selected as a 72 μm thick shell appressed with the implant surface (Supplementary Figure 2). Such ROI contained all cancellous bone in contact with implant surfaces, which could reflect the three-dimensional bone-to-implant contact. Then the BV/TV, BS/BV, Tb.Th, Tb.N, and Tb.Sp of each sample was calculated based on the ROI to detect the amount, morphology, trabecular thickness, trabecular separation, and trabecular number, respectively, according to the bone histomorphometry guide by The Histomorphometry Nomenclature Committee of The American Society for Bone and Mineral Research (Dempster et al., 2013).

#### Undecalcified Histology Sections

After micro CT scanning, bone blocks were dehydrated in gradient ethanol solutions and embedded in the light-curing embedding resin (Technovit 7200 VLC, Kulzer, Germany). Using an undecalcified hard tissue microtome (300CP, EXAKT, Germany), primary sections in 200 μm thickness were prepared firstly. Then, primary sections were grinded and polished to the final thickness of 20∼30 μm using a hard tissue grinding system (400CS, EXAKT, Germany). Finally, Toluidine Blue O was used for staining sections. The osteoid was labeled as light blue while mature bone was deeper blue (Mouraret et al., 2014). Using the Bioquant Osteo 2019 software (Bioquant, United States), the bone-to-implant contact (BIC) was calculated by the length ratio of the bone-to-implant contact zone and intrabony implant perimeter (Supplementary Figure 3A). Also, the volume and surface fraction of osteoid was quantified by OV/BV and OS/BS, in the wound chamber between adjacent intrabony implant threads (Supplementary Figure 3B; Berglundh et al., 2003; Dempster et al., 2013).

### Statistical Analysis

Data of qRT-PCR and bone histomorphometric analysis were expressed as mean ± standard deviation (SD). In cell experiments, three parallel samples in each group were used, and a multiple *t*-test with Benjamini, Krieger, and Yekutieli’s *post hoc* was used for analyzing the significant difference. For comparing the osseointegration of SLM-UT, SLM-CO, and TixOs implants at 2, 4, and 8 weeks, a two-way analysis of variance (two-way ANOVA) with Tukey’s *post hoc* was conducted in the animal experiment. The value of *P* < 0.05 was considered statistically significant.

## Results

### Surface Topography and Roughness

**FIGURE 1:**
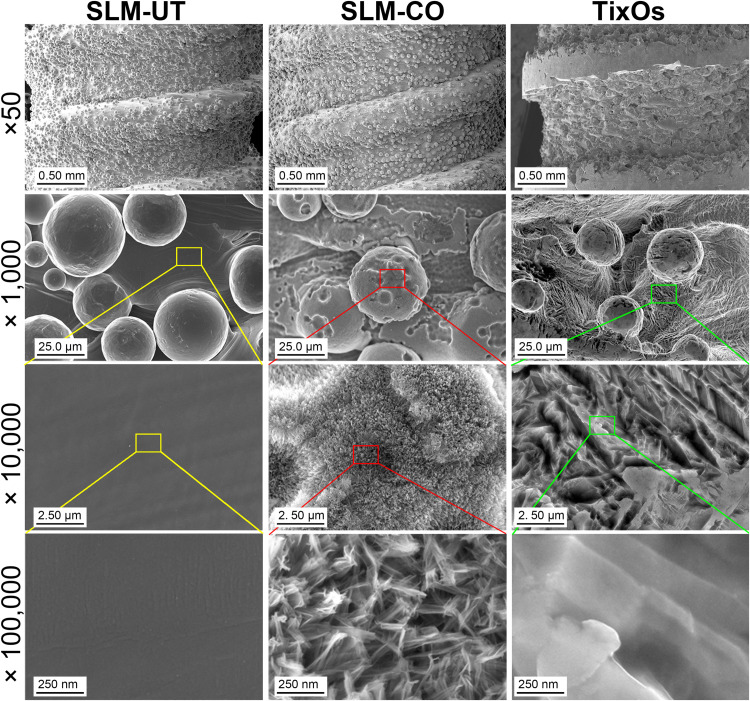
Surface topography of three implants by the field emission scanning electron microscope.

**FIGURE 2:**
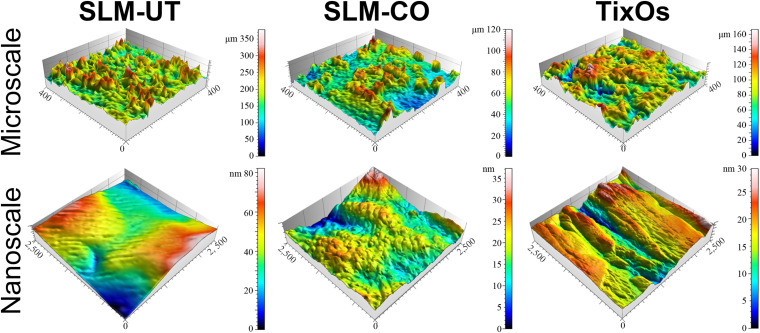
Surface three-dimensional reconstruction view of three implants by the profilometer (at microscale) and atomic force microscope (at nanoscale).

The surface topography of SLM-UT, SLM-CO, and TixOs implants under FE-SEM were shown in Figure 1. At low magnifications, surfaces of three SLM implants were rich in randomly distributed balling structures (diameter: 10∼30 μm). At higher magnifications, the surface of SLM-CO implants displayed nanoneedles (diameter: 20∼50 nm, height: 150∼250 nm). No specific nanostructure was observed on the surface of SLM-UT implants. Groove submicrostructures (width: 0.17∼2.17 μm) were observed on the surface of TixOs implants, without specific nanostructure either. Figure 2 illustrated the reconstructed 3D view of each implant by the profilometer and AFM, which was consistent with the FE-SEM observation. The S_*a*_ at micro and nano scales were calculated and listed in Table 2. The sequence of microroughness was SLM-UT>TixOs>SLM-CO, and of nanoroughness was SLM-CO > TixOs > SLM-UT.

**TABLE 2:** The S_a_ at micro and nano scale of three implants in this study.

	**S_a_ at microscale (μm)**	**S_a_ at nanoscale (nm)**
SLM-UT	28.28	7.57
SLM-CO	12.82	54.33
TixOs	19.49	31.67

### Surface Chemical Composition and Wettability

**FIGURE 3:**
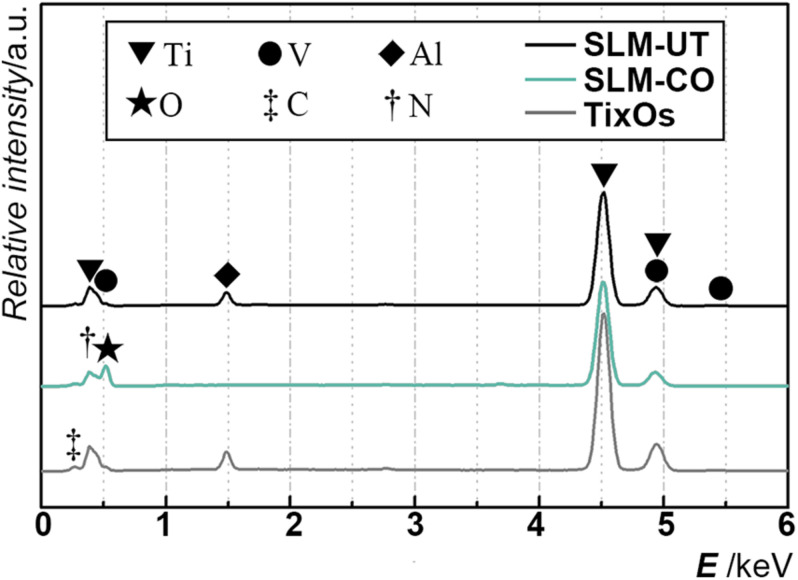
The elements component of three implants, illustrated by the multi-curve-stack lines by Y offsets graph of energy-dispersive X-ray spectrometry.

**FIGURE 4:**
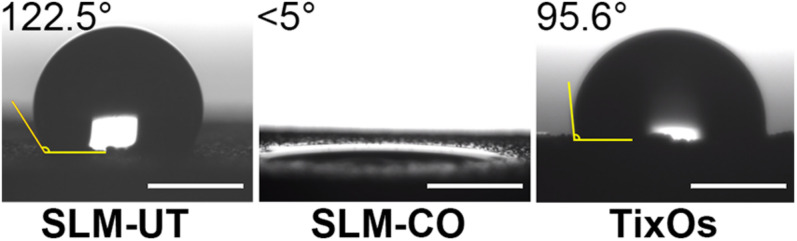
The contact angle in the air with distilled water of three implants (bar = 0.5 mm).

The multi-curve-stack lines by Y offsets of EDS showed the composition of the chemical elements (Figure 3). Ti, Al, and V were detected on the surface of SLM-UT implants. Ti, O, and N were detected on the surface of SLM-CO implants. Ti, Al, V, C, and O were detected on the surface of TixOs implants. The weight percentage of each element were listed in Table 3. A video of the wetting process showed enhanced wettability of nanomodified SLM titanium surfaces (Movie 1). Both distilled water and blood could spread over quickly on SLM-CO rather than keeping relative static on SLM-UT and TixOs surfaces. To describe this striking contrast quantitatively, Figure 4 showed the contact angle of three implants with distilled water in the air. The contact angle of SLM-UT and TixOs surfaces were higher than 90°, and of SLM-CO was less than 5°.

**TABLE 3:** The surface chemical composition (weight percentage) of three implants in this study.

	**Ti**	**O**	**Al**	**V**	**Other elements**
SLM-UT	89.4%	–	6.3%	4.3%	–
SLM-CO	63.0%	34.1%	–	–	2.9% N
TixOs	79.8%	4.5%	5.6%	4.6%	5.4% C

### Cell Proliferation and Osteogenesis *in vitro*

**FIGURE 5:**
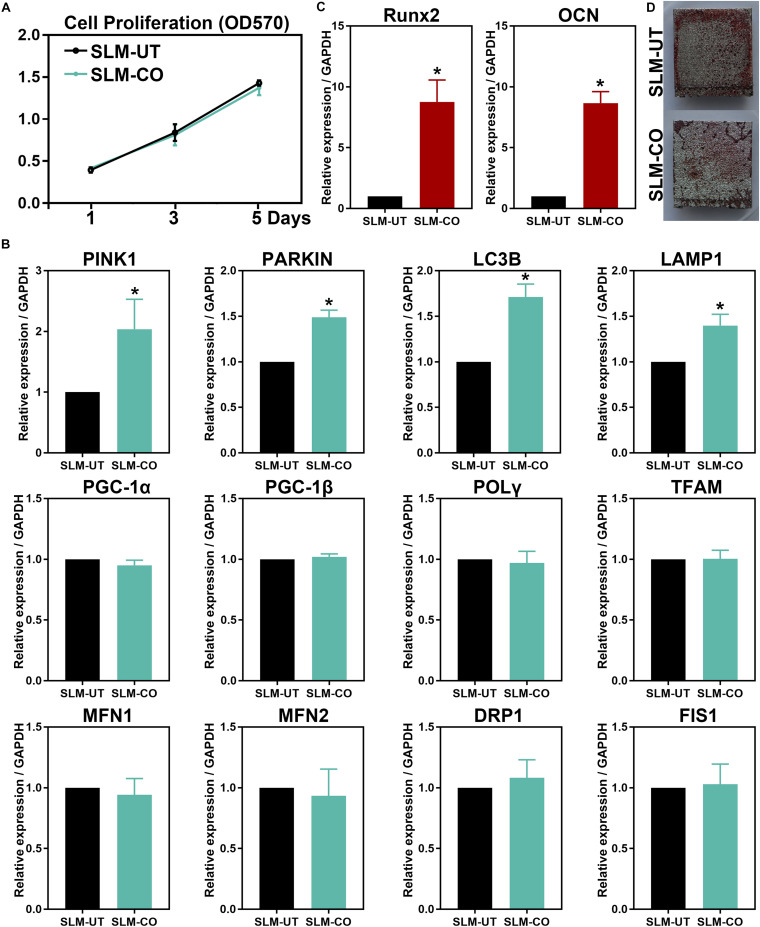
The MG-63 cells proliferation, osteogenesis, and mitochondrial dynamics. **(A)** No significant difference in cell proliferation between SLM-UT and SLM-CO surfaces. **(B)** Upregulated relative gene expression of Runx2 and OCN at 14 days. **(C)** More continuous mineralization nodules of the SLM-CO surface. **(D)** Upregulated mitophagy related gene expression of the SLM-CO surface (*P < 0.05). No significant difference existed in mitochondrial biogenesis (PGC-1α, PGC-1β, POLγ, and TFAM), fission (DRP1 and FIS1), and fusion (MFN1 and MFN2) related gene expression (*P* > 0.05).

The MG-63 cells proliferation measured by MTT assay was shown in Figure 5A. At 1, 3, and 5 days after cell seeding, the formazan solution absorbance of SLM-UT and SLM-CO surfaces both increased gradually. No significant difference existed between the two groups, implying that MG-63 cell proliferation was not affected on nanomodified SLM titanium surfaces.

At 5 days after cell seeding, significantly upregulated mitophagy markers (PINK1, PARKIN, LC3B, and LAMP1) of the SLM-CO surface were observed (Figure 5B). No significant difference was found on mitochondrial biogenesis (PGC-1α, PGC-1β, POLγ, and TFAM), fusion (MFN1 and MFN2), and fission (DRP1 and FIS1) markers between SLM-UT and SLM-CO surfaces. After culturing for 14 days, Figure 5C showed the relative expression level of Runx2 and OCN were upregulated significantly on SLM-CO surfaces. At the same time point, Figure 5D illustrated the photograph of alizarin red staining. The mineralized nodules on SLM-UT surfaces were sporadic and point-like, while on SLM-CO surfaces were larger and more continuous. These data indicated that the osteogenesis of MG-63 cells could be promoted by the nanomodified SLM titanium surfaces.

### Osseointegration *in vivo*

**FIGURE 6:**
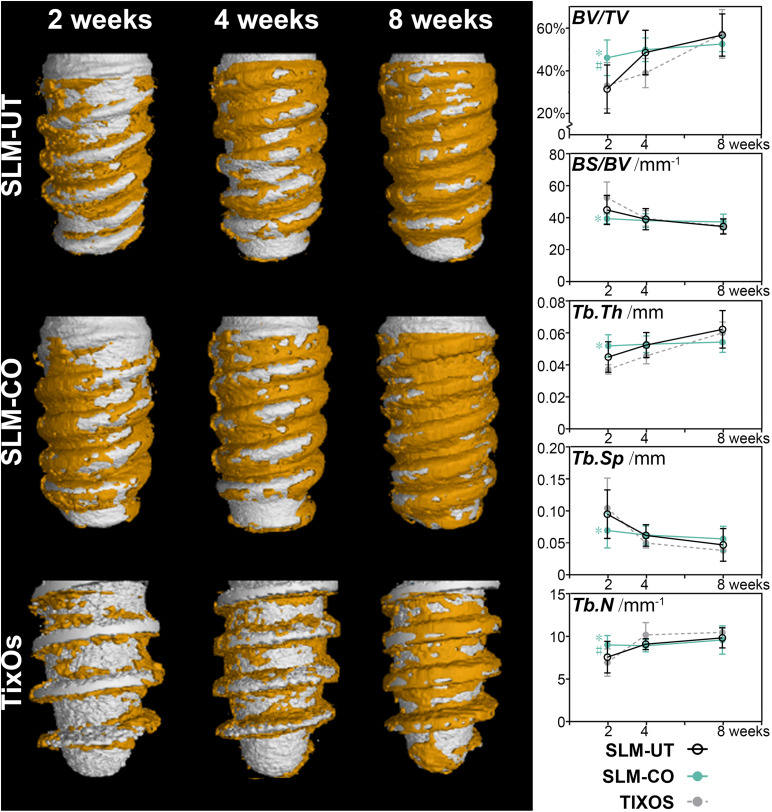
The time sequence of osseointegration showed by micro-CT and the corresponding quantitative analysis of bone structural parameters (*significant difference between SLM-CO and TixOs, *P* < 0.05, ^#^significant difference between SLM-CO and SLM-UT, *P* < 0.05).

The micro CT rendering graphic, accompanied with bone structural parameters of peri-implant bone were shown in Figure 6. The overall trend was the amount of peri-implant bone of three implants was gradually increasing from 2 to 8 weeks after surgical implantation. At the time point of 2 weeks, the peri-implant bone of SLM-CO implants demonstrated a higher amount and density than SLM-UT and TixOs implants. Quantitatively, the BV/TV, Tb.Th, and Tb.N of SLM-CO implants at 2 weeks were significantly higher than SLM-UT and TixOs implants. The BS/BV and Tb.Sp of SLM-CO implants at 2 weeks were significantly lower than SLM-UT and TixOs implants. At 4 and 8 weeks, no significant differences of bone structural parameters were found between surfaces of SLM-UT, SLM-CO, and TixOs implants.

**FIGURE 7:**
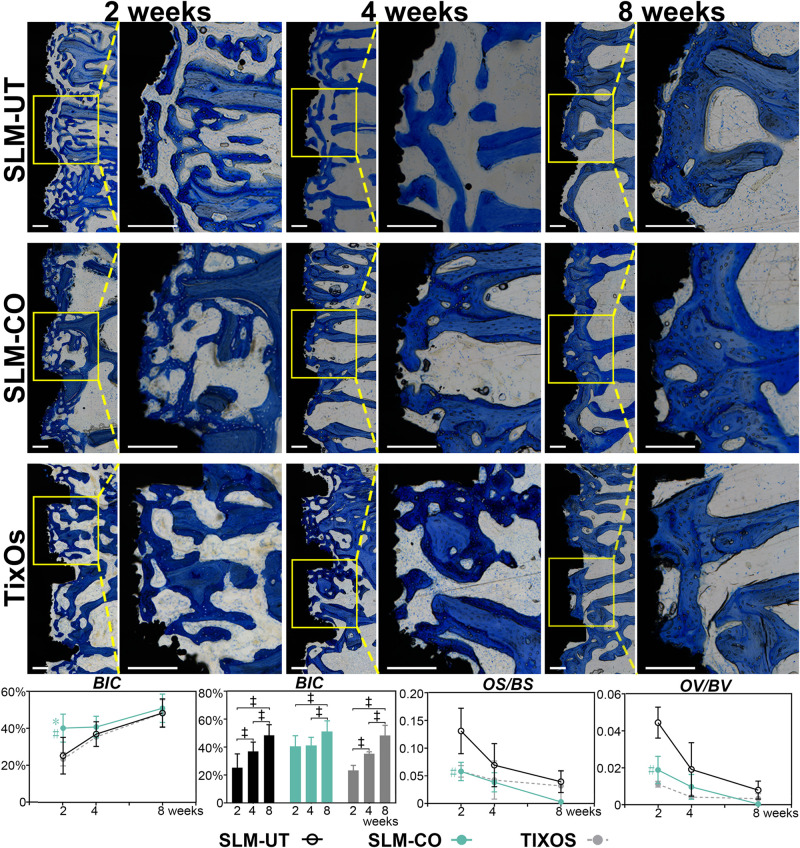
The time sequence of osseointegration showed by Toluidine Blue O stained undecalcified histological sections and the corresponding quantitative analysis of bone-to-implant contact (BIC) and osteoid content (^‡^significant difference of BIC between different time points, *P* < 0.05, *significant difference between SLM-CO and TixOs, *P* < 0.05, ^#^significant difference between SLM-CO and SLM-UT, *P* < 0.05).

The utmost advantages of histological sections were including tissue response information than micro CT (Bissinger et al., 2017). Hence, we further quantified the mineralization level of peri-implant bone by undecalcified histology sections. As Figure 7 illustrated, immature osteoid and woven bone at 2 weeks were gradually replaced by parallel-fiber bone, and finally transformed to mature lamellar bone at 8 weeks. The morphology of peri-implant bone was discrete and thin at 2 weeks, and become relatively continuous at 8 weeks. Quantitatively, the BIC of SLM-CO implants was significantly higher than SLM-UT and TixOs implants at 2 weeks and demonstrated no significant difference between 2 and 4 weeks. The most striking result was that the OV/BV and OS/BS of SLM-CO implants were significantly lower than SLM-UT implants at 2 weeks, which implied enhanced initial bone mineralization on nanomodified SLM dental implants. At 4 and 8 weeks, no significant differences of BIC, OV/BV, and OS/BS was found between SLM-UT, SLM-CO, and TixOs implants.

## Discussion

In this study, according to bone histomorphology, it was proved that the peri-implant bone formation and mineralization could be promoted by the hierarchical micro-nano topography based on SLM dental implants. Besides, the *in vitro* effects on mitochondrial dynamics and osteogenesis was investigated as a possible mechanism in enhancing osseointegration. Some issues will be discussed next.

The SLM was a layer-by-layer fabrication process that used a laser to melt and fuse specific regions of metal powder (Yap et al., 2015). Universally, the natural surface topography of SLM titanium implants was coarse balling structures with a diameter of tens of microns (Ahmed, 2019). In implant dentistry, it had been recommended that both micro and nano topography could enhance osseointegration. The major contribution of the microtopography was improving cell compatibility and bone-to-implant mechanical interlocking (Gittens et al., 2011; Albrektsson and Wennerberg, 2019), while the positive effect of the nanotopography was enhancing the initial adsorption of key proteins in tissue healing (Rupp et al., 2018; Albrektsson and Wennerberg, 2019; Ferraris et al., 2019). Combining advantages of microtopography and nanotopography, the hierarchical surface micro-nano topography had demonstrated its superiority in enhancing osseointegration of SLM titanium implants (Xu et al., 2016; Yu et al., 2018, 2019). This trend could also be revealed by enhanced osteogenic gene expression, BIC, and BV/TV in this study. The denser peri-implant bone of nanomodified SLM implants than untreated SLM and TixOs implants might contribute to strengthening the bone-to-implant mechanical anchorage. Osteoid was believed as the signal of new bone formation, and the amount of which decreased while new bone becoming mineralized and mature (Stadlinger et al., 2009; Ocana et al., 2017). We found less osteoid beside nanomodified SLM implants than untreated SLM implants at 2 weeks. Considering the higher BIC of nanomodified SLM titanium implants, this finding confirmed that the hierarchical surface micro-nano topography could promote peri-implant bone mineralization of SLM implants. No significant difference in osteoid content existed between the TixOs and SLM-CO group. A possible reason might be the submicron grooves on surfaces of TixOs implants could promote peri-implant bone mineralization. In conclusion, the histological evidence in this study could support the significance of hierarchical micro-nano topography on inorganic chemical oxidized SLM implants.

In cell experiments, the advantages of hierarchical micro-nano topography based on SLM titanium surfaces were also proven. Mitochondria acted as the potential target of nanomaterials and played an essential role in the energy metabolism of eukaryotic cells (Shen et al., 2018). Balanced mitochondrial dynamics could facilitate natural homeostasis of tissue function (Li et al., 2017). Detailly, mitochondrial biogenesis, fusion, and fission could keep mitochondria healthy, while fission and mitophagy could segregate and remove damaged mitochondria (Ni et al., 2015; Shen et al., 2018). Mitophagy was an evolutionarily conserved cellular process to stabilize cell energy metabolism by removing dysfunctional or superfluous mitochondria (Ashrafi and Schwarz, 2013; Palikaras et al., 2018). Also, the process of mitophagy was proven to participate in the cell-mediated biomineralization process. To be specific, the amorphous calcium phosphate, which was the precursor in biomineralization, could trigger mitophagy to transport intracellularly *via* the autophagosome and released to the extracellular matrix *via* exocytosis (Pei et al., 2018). In this study, with the relative expression of mitophagy-related gene upregulated at 5 days, the osteogenesis-related gene expression of SLM-CO group was promoted, which was consistent with other relevant studies (Hyzy et al., 2016; H. Wang et al., 2018; Yu et al., 2018). These results confirmed our conjecture that the hierarchical micro-nano topography could influence cell bioactivity for better osteogenesis performance.

Implant surface modifications were closely influencing osseointegration (Bosshardt et al., 2017). Physical, chemical, or biological techniques were used in surface modification of dental implants (Liu et al., 2019). In general, controlling surface roughness has been the holy grail of implant surface modification, avoiding limitations on detachment or potential toxicity of extra materials coating (Modaresifar et al., 2019). To form hierarchical micro-nano topography, plenty of techniques had been used in increasing the nanoroughness of microrough SLM titanium implants. Anodization was to induce nanopores or nanotubes on the anode metal surface under the assistance of electric field and temperature (Minagar et al., 2012; Q. Wang et al., 2020). The other process, hydrothermal treatment, was to prepare titanium oxide layer with nanotopography *via* surface crystallization procedure within an elevated temperature and aqueous media (Ali et al., 2020; Bencina et al., 2020). However, these techniques strongly depended on specific apparatus like electrochemical reactors or tefion-lined autoclaves. Lately, an inorganic chemical modification protocol was investigated, which simply used hydrofluoride acid and peroxide hydrogen to create nanotopography on titanium surfaces (Ferraris et al., 2011). Such modification had been demonstrated to improve osteoblasts progenitors’ adhesion, proliferation, and extracellular matrix deposition of additive manufactured titanium implants (Ferraris et al., 2019), but its *in vivo* osseointegration had not been reported yet. In this study, we utilized an improved protocol of inorganic chemical oxidation to create nanotopography on SLM Ti-6Al-4V implant surfaces. Without expensive equipment and dangerous reagents, using hydrochloride acid and peroxide hydrogen at room temperature was relatively safe and low-cost. The surface topography of SLM-CO implants was similar to which of hydrothermal treated (Xu et al., 2016; Yu et al., 2018) or inorganic chemical oxidized (Ferraris et al., 2011) SLM implants in previous studies. Moreover, these nanoneedles could form extracellular matrix-like nanonets. Such nanonets structure had been proven to promote the alkaline phosphatase activity in osteogenesis than anodized nanotubes on SLM titanium surfaces (Xu et al., 2016). The relative higher value of nano-S_*a*_ could also reflect the existence of nanotopography of SLM-CO implants.

Considering that almost all surface modifications could alter the surface chemical and physical character (Dohan Ehrenfest et al., 2011; Albrektsson and Wennerberg, 2019), we further measured chemical element components and contact angle of different surfaces. The surface chemical elements component of SLM-CO implants was close to that of other nanomodified SLM titanium surfaces modified by anodization (64.11% titanium and 29.50% oxygen) and hydrothermal treatment (51.69% titanium and 43.53% oxygen) (Xu et al., 2016). Also, the absence of aluminum and vanadium on the SLM-CO surface kept consistence with anodized and hydrothermal treated SLM surface (Xu et al., 2016), which implied the formation of titanium oxide layer. Moreover, 2.9% nitrogen was found at the surface of SLM-CO implants, which was less than the impurity content of TixOs implants. The surface hydrophilicity was an important physical character influenced by surface modification, which could promote the initial blood wetting, protein adsorption, and subsequent wound healing process (Minagar et al., 2013; Chambrone et al., 2015; Calciolari et al., 2018; Donos et al., 2018; Kitajima et al., 2020). Generally, the osteogenesis stimulating effect of hydrophilic titanium surfaces was suggested (Gittens et al., 2014; Rupp et al., 2018). While the contact angle of SLM-UT and TixOs implants were similar to which of pure titanium (about 90°(Gittens et al., 2014)), the contact angle of SLM-CO implants was less than 5° and close to which of other nanomodified SLM implants (Hyzy et al., 2016). These results implied the benign chemical and physical characteristics of hierarchical surface micro-nano topography on SLM-CO implants.

In general, peri-implant new bone formation and mineralization of SLM titanium implants were successfully enhanced by the hierarchical micro-nano topography. The up-regulation of mitophagy level might serve as the facilitation. Two possible issues should be investigated in further study. Firstly, the enhancement of mitophagy was only studied at the gene expression level in the current study. Future works should be organized with more molecular biological methods. Secondly, the results of animal experiments on New Zealand rabbits could not be applied to clinical treatments. The human osseointegration of nanomodified SLM dental implants still needs to be evaluated through rigorous clinical trials and compared with conventional dental implants.

## Data Availability Statement

The original contributions presented in the study are included in the article/Supplementary Material, further inquiries can be directed to the corresponding authors.

## Ethics Statement

The animal study was reviewed and approved by The Laboratory Animal Care and Welfare Committee, Fourth Military Medical University.

## Author Contributions

DP and AL provided the original research idea and constructive guidance. TS, YZ, YC, and GH fabricated the materials. TS, YZ, GS, and YP performed the experiments and organized the data. TS, YZ, GS, YC, GH, DP, and AL interpreted the data and wrote the manuscript. AL provided the financial support for this work. All authors contributed to the article and approved the submitted version.

## Conflict of Interest

The authors declare that the research was conducted in the absence of any commercial or financial relationships that could be construed as a potential conflict of interest. The handling editor declared a past co-authorship with one of the authors YC.

## References

[B1] Ahmed N. (2019). Direct metal fabrication in rapid prototyping: a review. *J. Manufact. Process.* 42 167–191. 10.1016/j.jmapro.2019.05.001

[B2] Albrektsson T. Wennerberg A. (2019). On osseointegration in relation to implant surfaces. *Clin. Implant. Dent. Relat. Res.* 21(Suppl. 1) 4–7. 10.1111/cid.12742 30816639

[B3] Ali N. Ali F. Khurshid R. Ikramullah, Ali Z. Afzal A. (2020). TiO2 nanoparticles and Epoxy-TiO2 nanocomposites: a review of synthesis, modification strategies, and photocatalytic potentialities. *J. Inorgan. Organometal. Polym. Mater.* 30 4829–4846. 10.1007/s10904-020-01668-6

[B4] Andani M. T. Shayesteh Moghaddam N. Haberland C. Dean D. Miller M. J. Elahinia M. (2014). Metals for bone implants. Part 1. Powder metallurgy and implant rendering. *Acta Biomater.* 10 4058–4070. 10.1016/j.actbio.2014.06.025 24956564

[B5] Ashrafi G. Schwarz T. L. (2013). The pathways of mitophagy for quality control and clearance of mitochondria. *Cell Death Differ.* 20 31–42. 10.1038/cdd.2012.81 22743996 PMC3524633

[B6] Bencina M. Iglic A. Mozetic M. Junkar I. (2020). Crystallized TiO2 nanosurfaces in biomedical applications. *Nanomaterials (Basel)* 10:1121. 10.3390/nano10061121 32517276 PMC7353402

[B7] Berglundh T. Abrahamsson I. Lang N. P. Lindhe J. (2003). De novo alveolar bone formation adjacent to endosseous implants. *Clin. Oral Implants Res.* 14 251–262. 10.1034/j.1600-0501.2003.00972.x 12755774

[B8] Bhargav A. Sanjairaj V. Rosa V. Feng L. W. Fuh Yh J. (2018). Applications of additive manufacturing in dentistry: a review. *J. Biomed. Mater. Res. B Appl. Biomater.* 106 2058–2064. 10.1002/jbm.b.33961 28736923

[B9] Bissinger O. Probst F. A. Wolff K. D. Jeschke A. Weitz J. Deppe H. (2017). Comparative 3D micro-CT and 2D histomorphometry analysis of dental implant osseointegration in the maxilla of minipigs. *J. Clin. Periodontol.* 44 418–427. 10.1111/jcpe.12693 28063250

[B10] Bosshardt D. D. Chappuis V. Buser D. (2017). Osseointegration of titanium, titanium alloy and zirconia dental implants: current knowledge and open questions. *Periodontology 2000* 73 22–40. 10.1111/prd.12179 28000277

[B11] Buser D. Sennerby L. De Bruyn H. (2017). Modern implant dentistry based on osseointegration: 50 years of progress, current trends and open questions. *Periodontology 2000* 73 7–21. 10.1111/prd.12185 28000280

[B12] Calciolari E. Hamlet S. Ivanovski S. Donos N. (2018). Pro-osteogenic properties of hydrophilic and hydrophobic titanium surfaces: Crosstalk between signalling pathways in in vivo models. *J. Periodont. Res.* 53 598–609. 10.1111/jre.12550 29687451

[B13] Chambrone L. Shibli J. A. Mercurio C. E. Cardoso B. Preshaw P. M. (2015). Efficacy of standard (SLA) and modified sandblasted and acid-etched (SLActive) dental implants in promoting immediate and/or early occlusal loading protocols: a systematic review of prospective studies. *Clin. Oral Implants Res.* 26 359–370. 10.1111/clr.12347 24814519

[B14] Cui Y. Zhu T. Li D. Li Z. Leng Y. Ji X. (2019). Bisphosphonate-functionalized scaffolds for enhanced bone regeneration. *Adv. Healthc Mater.* 8:e1901073. 10.1002/adhm.201901073 31693315

[B15] Dempster D. W. Compston J. E. Drezner M. K. Glorieux F. H. Kanis J. A. Malluche H. (2013). Standardized nomenclature, symbols, and units for bone histomorphometry: a 2012 update of the report of the ASBMR Histomorphometry Nomenclature Committee. *J. Bone Miner. Res.* 28 2–17. 10.1002/jbmr.1805 23197339 PMC3672237

[B16] Dohan Ehrenfest D. M. Vazquez L. Park Y. J. Sammartino G. Bernard J. P. (2011). Identification card and codification of the chemical and morphological characteristics of 14 dental implant surfaces. *J. Oral Implantol.* 37 525–542. 10.1563/AAID-JOI-D-11-00080 21728785

[B17] Donos N. Horvath A. Mezzomo L. A. Dedi D. Calciolari E. Mardas N. (2018). The role of immediate provisional restorations on implants with a hydrophilic surface: a randomised, single-blind controlled clinical trial. *Clin. Oral Implants Res.* 29 55–66. 10.1111/clr.13038 28833613

[B18] Dos Santos L. C. P. Malheiros F. C. Guarato A. Z. (2019). Surface parameters of as-built additive manufactured metal for intraosseous dental implants. *J. Prosthet. Dent.* 124 217–222. 10.1016/j.prosdent.2019.09.010 31759564

[B19] Ferraris S. Cochis A. Cazzola M. Tortello M. Scalia A. Spriano S. (2019). Cytocompatible and anti-bacterial adhesion nanotextured titanium oxide layer on titanium surfaces for dental and orthopedic implants. *Front. Bioeng. Biotechnol.* 7:103. 10.3389/fbioe.2019.00103 31143762 PMC6520600

[B20] Ferraris S. Spriano S. Pan G. Venturello A. Bianchi C. L. Chiesa R. (2011). Surface modification of Ti-6Al-4V alloy for biomineralization and specific biological response: Part I, inorganic modification. *J. Mater. Sci. Mater. Med.* 22 533–545. 10.1007/s10856-011-4246-2 21287240

[B21] Gittens R. A. McLachlan T. Olivares-Navarrete R. Cai Y. Berner S. Tannenbaum R. (2011). The effects of combined micron-/submicron-scale surface roughness and nanoscale features on cell proliferation and differentiation. *Biomaterials* 32 3395–3403. 10.1016/j.biomaterials.2011.01.029 21310480 PMC3350795

[B22] Gittens R. A. Scheideler L. Rupp F. Hyzy S. L. Geis-Gerstorfer J. Schwartz Z. (2014). A review on the wettability of dental implant surfaces II: Biological and clinical aspects. *Acta Biomater.* 10 2907–2918. 10.1016/j.actbio.2014.03.032 24709541 PMC4103435

[B23] Gulati K. Prideaux M. Kogawa M. Lima-Marques L. Atkins G. J. Findlay D. M. (2017). Anodized 3D-printed titanium implants with dual micro- and nano-scale topography promote interaction with human osteoblasts and osteocyte-like cells. *J. Tissue Eng. Regen. Med.* 11 3313–3325. 10.1002/term.2239 27925441

[B24] Hyzy S. L. Cheng A. Cohen D. J. Yatzkaier G. Whitehead A. J. Clohessy R. M. (2016). Novel hydrophilic nanostructured microtexture on direct metal laser sintered Ti-6Al-4V surfaces enhances osteoblast response in vitro and osseointegration in a rabbit model. *J. Biomed. Mater. Res. A* 104 2086–2098. 10.1002/jbm.a.35739 27086616

[B25] Kitajima H. Hirota M. Iwai T. Hamajima K. Ozawa R. Hayashi Y. (2020). Computational fluid simulation of fibrinogen around dental implant surfaces. *Int. J. Mol. Sci.* 21:660. 10.3390/ijms21020660 31963895 PMC7014059

[B26] Li Q. Gao Z. Chen Y. Guan M. X. (2017). The role of mitochondria in osteogenic, adipogenic and chondrogenic differentiation of mesenchymal stem cells. *Protein Cell* 8 439–445. 10.1007/s13238-017-0385-7 28271444 PMC5445026

[B27] Liu Y. Rath B. Tingart M. Eschweiler J. (2019). Role of implants surface modification in osseointegration: a systematic review. *J. Biomed. Mater. Res. A* 108 470–484. 10.1002/jbm.a.36829 31664764

[B28] Mangano C. Mangano F. G. Shibli J. A. Ricci M. Perrotti V. d’Avila S. (2012). Immediate loading of mandibular overdentures supported by unsplinted direct laser metal-forming implants: results from a 1-year prospective study. *J. Periodontol.* 83 70–78. 10.1902/jop.2011.110079 21627459

[B29] Mangano F. Mangano C. Piattelli A. Iezzi G. (2017). Histological evidence of the osseointegration of fractured direct metal laser sintering implants retrieved after 5 years of function. *Biomed. Res. Int.* 2017:9732136. 10.1155/2017/9732136 28929117 PMC5592009

[B30] Minagar S. Berndt C. C. Wang J. Ivanova E. Wen C. (2012). A review of the application of anodization for the fabrication of nanotubes on metal implant surfaces. *Acta Biomater.* 8 2875–2888. 10.1016/j.actbio.2012.04.005 22542885

[B31] Minagar S. Wang J. Berndt C. C. Ivanova E. P. Wen C. (2013). Cell response of anodized nanotubes on titanium and titanium alloys. *J. Biomed. Mater. Res. A* 101 2726–2739. 10.1002/jbm.a.34575 23436766

[B32] Modaresifar K. Azizian S. Ganjian M. Fratila-Apachitei L. E. Zadpoor A. A. (2019). Bactericidal effects of nanopatterns: a systematic review. *Acta Biomater.* 83 29–36. 10.1016/j.actbio.2018.09.059 30273746

[B33] Mouraret S. Hunter D. J. Bardet C. Brunski J. B. Bouchard P. Helms J. A. (2014). A pre-clinical murine model of oral implant osseointegration. *Bone* 58 177–184. 10.1016/j.bone.2013.07.021 23886841 PMC4962868

[B34] Ni H. M. Williams J. A. Ding W. X. (2015). Mitochondrial dynamics and mitochondrial quality control. *Redox Biol.* 4 6–13. 10.1016/j.redox.2014.11.006 25479550 PMC4309858

[B35] Ocana R. P. Rabelo G. D. Sassi L. M. Rodrigues V. P. Alves F. A. (2017). Implant osseointegration in irradiated bone: an experimental study. *J. Periodont. Res.* 52 505–511. 10.1111/jre.12416 27624290

[B36] Oliveira T. T. Reis A. C. (2019). Fabrication of dental implants by the additive manufacturing method: a systematic review. *J. Prosthet. Dent.* 122 270–274. 10.1016/j.prosdent.2019.01.018 30928226

[B37] Palikaras K. Lionaki E. Tavernarakis N. (2018). Mechanisms of mitophagy in cellular homeostasis, physiology and pathology. *Nat. Cell Biol.* 20 1013–1022. 10.1038/s41556-018-0176-2 30154567

[B38] Pei D. D. Sun J. L. Zhu C. H. Tian F. C. Jiao K. Anderson M. R. (2018). Contribution of mitophagy to cell-mediated mineralization: revisiting a 50-year-old conundrum. *Adv. Sci.* 5:1800873. 10.1002/advs.201800873 30356983 PMC6193168

[B39] Rupp F. Liang L. Geis-Gerstorfer J. Scheideler L. Huttig F. (2018). Surface characteristics of dental implants: a review. *Dent. Mater.* 34 40–57. 10.1016/j.dental.2017.09.007 29029850

[B40] Shen Y. Wu L. Qin D. Xia Y. Zhou Z. Zhang X. (2018). Carbon black suppresses the osteogenesis of mesenchymal stem cells: the role of mitochondria. *Part. Fibre Toxicol.* 15:16. 10.1186/s12989-018-0253-5 29650039 PMC5897950

[B41] Stadlinger B. Lode A. T. Eckelt U. Range U. Schlottig F. Hefti T. (2009). Surface-conditioned dental implants: an animal study on bone formation. *J. Clin. Periodontol.* 36 882–891. 10.1111/j.1600-051X.2009.01466.x 19735467

[B42] Trevisan F. Calignano F. Aversa A. Marchese G. Lombardi M. Biamino S. (2018). Additive manufacturing of titanium alloys in the biomedical field: processes, properties and applications. *J. Appl. Biomater. Funct. Mater.* 16 57–67. 10.5301/jabfm.5000371 28967051

[B43] Vos T. Allen C. Arora M. Barber R. Bhutta Z. Brown A. (2016). Global, regional, and national incidence, prevalence, and years lived with disability for 310 diseases and injuries, 1990–2015: a systematic analysis for the Global Burden of Disease Study 2015. *Lancet* 388 1545–1602. 10.1016/S0140-6736(16)31678-6 27733282 PMC5055577

[B44] Wancket L. M. (2015). Animal models for evaluation of bone implants and devices: comparative bone structure and common model uses. *Vet. Pathol.* 52 842–850. 10.1177/0300985815593124 26163303

[B45] Wang H. Zhang X. Wang H. Zhang J. Li J. Ruan C. (2018). Enhancing the osteogenic differentiation and rapid osseointegration of 3D printed Ti6Al4V implants via nano-topographic modification. *J. Biomed. Nanotechnol.* 14 707–715. 10.1166/jbn.2018.2551 31352944

[B46] Wang Q. Zhou P. Liu S. Attarilar S. Ma R. L. Zhong Y. (2020). Multi-scale surface treatments of titanium implants for rapid osseointegration: a review. *Nanomaterials (Basel)* 10:1244 10.3390/nano10061244 PMC7353126 32604854

[B47] Wu Y. Feng F. Xin H. Li K. Tang Z. Guo Y. (2019). Fracture strength and osseointegration of an ultrafine-grained titanium mini dental implant after macromorphology optimization. *ACS Biomater. Sci. Eng.* 5 4122–4130. 10.1021/acsbiomaterials.9b00406 33448813

[B48] Xu J. Y. Chen X. S. Zhang C. Y. Liu Y. Wang J. Deng F. L. (2016). Improved bioactivity of selective laser melting titanium: Surface modification with micro-/nano-textured hierarchical topography and bone regeneration performance evaluation. *Mater. Sci. Eng. C Mater. Biol. Appl.* 68 229–240. 10.1016/j.msec.2016.05.096 27524017

[B49] Yap C. Y. Chua C. K. Dong Z. L. Liu Z. H. Zhang D. Q. Loh L. E. (2015). Review of selective laser melting: materials and applications. *Appl. Phys. Rev.* 2:041101 10.1063/1.4935926

[B50] Yu M. Lin Y. Liu Y. Zhou Y. Liu C. Dong L. (2018). Enhanced osteointegration of hierarchical structured 3D-printed titanium implants. *ACS Appl. Bio Mater.* 1 90–99. 10.1021/acsabm.8b00017

[B51] Yu M. Liu Y. Yu X. Li J. Zhao W. Hu J. (2019). Enhanced osteogenesis of quasi-three-dimensional hierarchical topography. *J. Nanobiotechnol.* 17:102. 10.1186/s12951-019-0536-5 31581945 PMC6777029

[B52] Zhang Y. B. Liu X. C. Zeng L. D. Zhang J. Zuo J. L. Zou J. (2019). Polymer fiber scaffolds for bone and cartilage tissue engineering. *Adv. Funct. Mater.* 29:20 10.1002/adfm.201903279

[B53] Zheng H. Tian Y. Gao Q. Yu Y. Xia X. Feng Z. (2020). Hierarchical micro-nano topography promotes cell adhesion and osteogenic differentiation via integrin alpha2-PI3K-AKT signaling axis. *Front. Bioeng. Biotechnol.* 8:463. 10.3389/fbioe.2020.00463 32509748 PMC7248375

