# Supplementary material for: Enhanced Osseointegration by the Hierarchical Micro-Nano Topography on Selective Laser Melting Ti-6Al-4V Dental Implants
Source: Front Bioeng Biotechnol. 2021 Jan 7;8:621601. doi: 10.3389/fbioe.2020.621601 (PMC7817818; doi:10.3389/fbioe.2020.621601)
Supplement: Supplementary file 1 [file Data_Sheet_1.DOCX]

Supplementary Materials


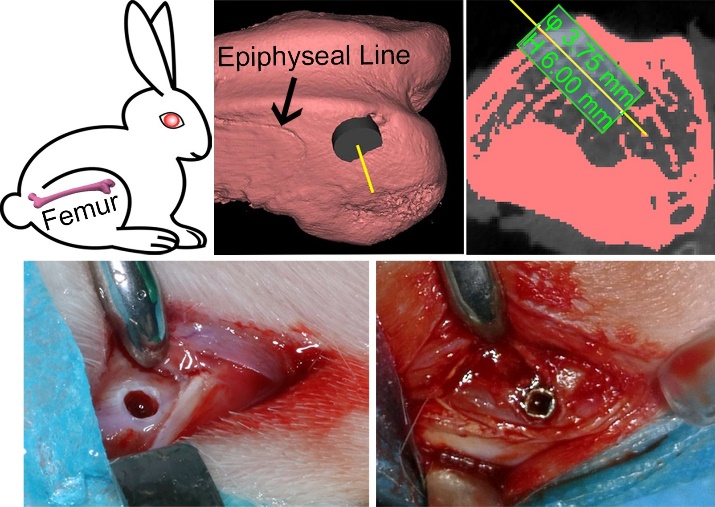


**Supplementary Figure 1.** The implant position and surgical incision.


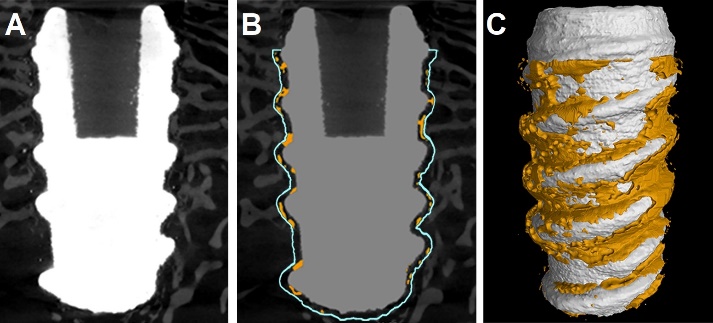


**Supplementary Figure 2.** (A) The axial section of the implant and peri-implant bone under micro CT scanning. (B) The region of interest for bone structural analysis (circled by the cyan line, on the view of A). (C) The three-dimensional rendering view of the implant (gray) and peri-implant bone (yellow).


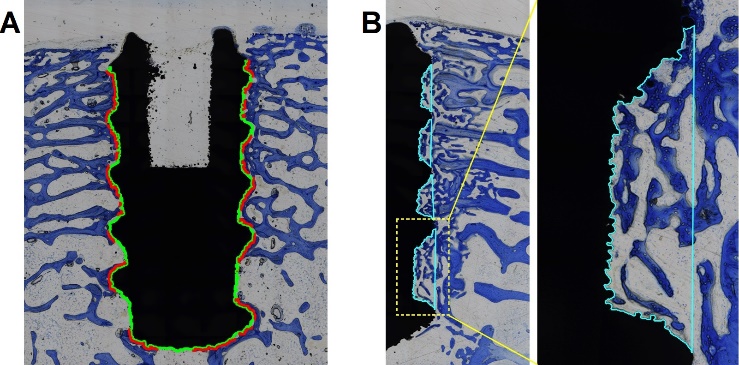


**Supplementary Figure 3.** (A) The calculation of bone-to-implant contact. Red: bone-to-implant contact zone. Green: intrabony implant perimeter. (B) The wound chamber for quantify osteoid (inside the cyan line).
